# Supplementary figures and images for: Upcycling of groundwater treatment sludge to magnetic Fe/Mn-bearing nanorod for chromate adsorption from wastewater treatment
Source: PLoS One. 2020 Jun 10;15(6):e0234136. doi: 10.1371/journal.pone.0234136 (PMC7286529; doi:10.1371/journal.pone.0234136)

**Graphic picture:**


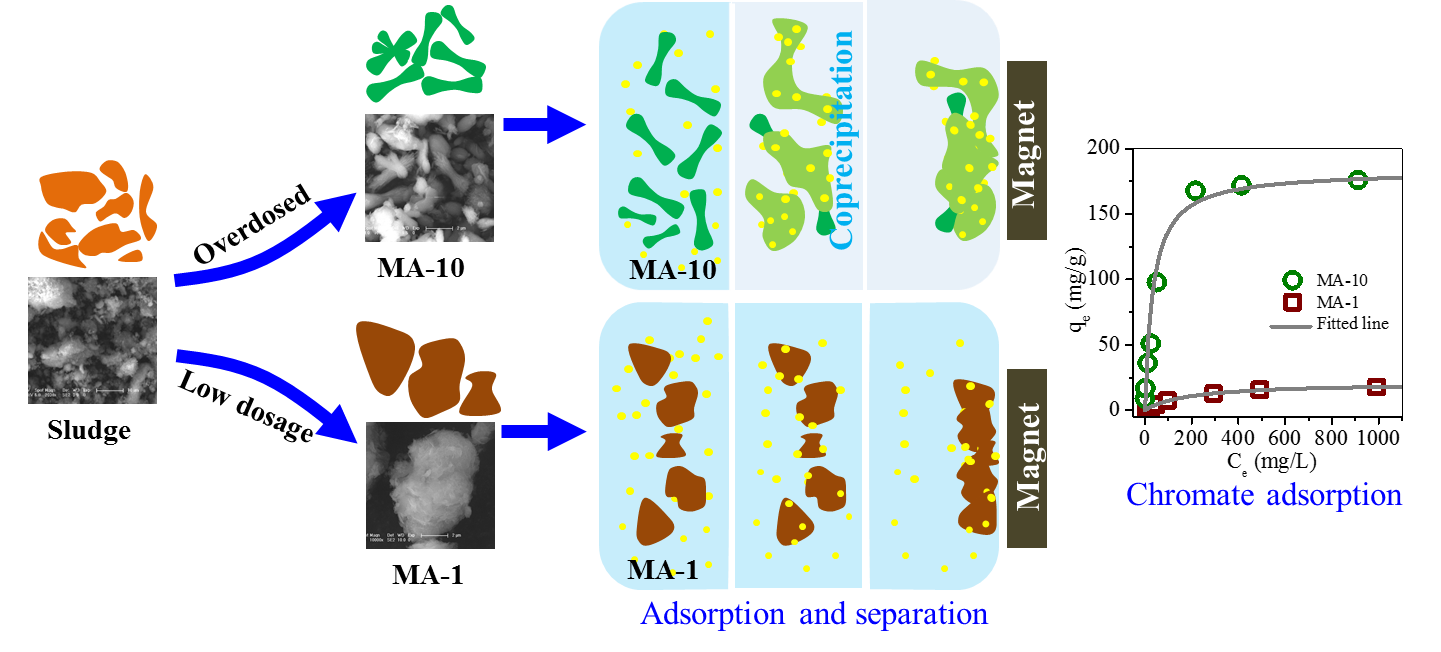

Supplement: S1 Data — (DOCX) [file pone.0234136.s001.docx]
