# Supplementary material for: Upcycling of groundwater treatment sludge to magnetic Fe/Mn-bearing nanorod for chromate adsorption from wastewater treatment
Source: PLoS One. 2020 Jun 10;15(6):e0234136. doi: 10.1371/journal.pone.0234136 (PMC7286529; doi:10.1371/journal.pone.0234136)
Supplement: S2 Data — (DOCX) [file pone.0234136.s002.docx]

Supplementary files

Related method

The composition of sludge, MA-1 and MA-10 was determined by X-ray fluorescence spectroscopy (S4-Explorer, Bruker, XRF, Germany).The X-ray powder diffraction (XRD) patterns of the sludge and the two MAs were determined using a diffractometry system (D/maxZ200PC, Rigaku, Japan) with copper (Cu) K a radiation in the 2θ range of 20°–50°. The crystallisation degree of the two MAs was analysed using Materials Studio software (V 6.0, Accelrys, USA) on the basis of the intensity and width of the standard peak of crystalline ferrihydrite phase compared with the background peaks. The crystalline phase of the Fe oxides in the sludge and the two MAs was investigated with a transmission Mössbauer spectrometer (MS-500, Oxford Instruments, UK) at room temperature. Magnetic measurement was conducted at room temperature by magnetometry (SQUID-VSM, Quantum Design, USA) with a SQUID-VSM system. The valence states of Mn on the sludge and MA surfaces were determined by X-ray photoelectron spectrometry (XPS, VG-ADES, Thermal VG, UK) with Mg K a radiation. Particle morphologies were observed with a field emission scanning electron microscope (FE-Nano SEM 450, FEI Co., USA) using a working voltage of 200 kV.
