# Supplementary material for: Upcycling of groundwater treatment sludge to magnetic Fe/Mn-bearing nanorod for chromate adsorption from wastewater treatment
Source: PLoS One. 2020 Jun 10;15(6):e0234136. doi: 10.1371/journal.pone.0234136 (PMC7286529; doi:10.1371/journal.pone.0234136)
Supplement: Attachment — Submitted filename: review1.docx [file pone.0234136.s003.docx]

**PONE-D-19-34495**

**Upcycling of groundwater treatment sludge to magnetic Fe/Mn-bearing nanorod for**

**chromate adsorption from wastewater treatment**

Dear Editor,

In this article “**Upcycling of groundwater treatment sludge to magnetic Fe/Mn-bearing nanorod for chromate adsorption from wastewater treatment” the author has investigated chromate adsorption from wastewater using sludge derived magnetic Fe/Mn-bearing nanorods.** The subject is interesting and cover the journal aims. Analysis and characterization of the composite may need detail discussion. The current version of the paper is not acceptable and need major revision.

The observations are as follows

1. The novelty of the work should be highlighted.
2. XRF analysis need detail explanation as it is missing in result and discussion part.
3. How the author confirms that the magnetic Fe/Mn-bearing nanorod derived from sludge is not toxic, some toxicity test/leachability test must be conducted to ensure nontoxicity of magnetic Fe/Mn-bearing nanorod in water.
4. In abstract and conclusion, it is mentioned that “This MA-10 showed 183.2 mg/g of chromate adsorption capacity” but in result and discussion the adsorption capacity is not discussed. The detail experiment explaining adsorption capacity must be discussed.
5. The advantage of taking magnetic adsorbent for chromate adsorption must be added in the introduction.
6. Table 2 shows Adsorption capacity of chromate by MA-10 is highest at pH2 but in section 2.3 the pH is mentioned 4.
7. Fig. 6 the scale of SEM images is not clear. How the author claims the composition of

Fe/Mn nanorods.

8. The results have not been explored in enough depth. Comprehensive discussion of the findings is completely missing from the result discussion section.
9. The conclusion should be in more detail.
